# Hepatitis B Virus Adaptation to the CD8+ T Cell Response: Consequences for Host and Pathogen

**DOI:** 10.3389/fimmu.2018.01561

**Published:** 2018-07-16

**Authors:** Sheila F. Lumley, Anna L. McNaughton, Paul Klenerman, Katrina A. Lythgoe, Philippa C. Matthews

**Affiliations:** ^1^Medawar Building for Pathogen Research, Nuffield Department of Medicine, University of Oxford, Oxford, United Kingdom; ^2^Department of Infectious Diseases and Microbiology, Oxford University Hospitals NHS Foundation Trust, John Radcliffe Hospital, Oxford, United Kingdom; ^3^Oxford BRC, John Radcliffe Hospital, Oxford, United Kingdom; ^4^Nuffield Department of Medicine, Big Data Institute, University of Oxford, Oxford, United Kingdom

**Keywords:** hepatitis B virus, evolution, adaptation, diversity, CD8+ T cells, adaptive immunity, human leukocyte antigen

## Abstract

Chronic viral hepatitis infections are a major public health concern, with an estimated 290 million individuals infected with hepatitis B virus (HBV) globally. This virus has been a passenger in human populations for >30,000 years, and remains highly prevalent in some settings. In order for this endemic pathogen to persist, viral adaptation to host immune responses is pre-requisite. Here, we focus on the interplay between HBV infection and the CD8+ T cell response. We present the evidence that CD8+ T cells play an important role in control of chronic HBV infection and that the selective pressure imposed on HBV through evasion of these immune responses can potentially influence viral diversity, chronicity, and the outcome of infection, and highlight where there are gaps in current knowledge. Understanding the nature and mechanisms of HBV evolution and persistence could shed light on differential disease outcomes, including cirrhosis and hepatocellular carcinoma, and help reach the goal of global HBV elimination by guiding the design of new strategies, including vaccines and therapeutics.

## Introduction

Within hosts, viruses with high mutation rates can rapidly adapt to the selection pressures placed upon them, including natural and vaccine induced immune responses, and antiviral therapy. Hepatitis B virus (HBV) represents a substantial international public health challenge, with an estimated 290 million people chronically infected globally (1). In this review, we explore the evidence for HBV escape from the CD8+ T cell response and examine the influence this process could have on infection outcomes.

**Figure 1:**
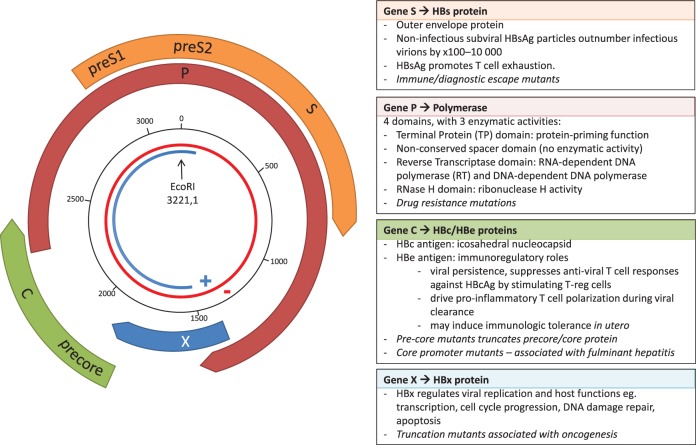
The overlapping genome structure of hepatitis B virus (HBV). The partially double-stranded circular DNA genome is shown with the negative strand in red and the positive strand in blue. The black numbered circle indicates the nucleotide position. The four genes, X, Polymerase (P), Core (C), and Surface (S) are represented by the thick colored arrows, with the main functional roles of the four genes shown in the boxes, and key impact of mutations shown in italics.

Hepatitis B virus belongs to the Hepadnaviridae family of small, enveloped, primarily hepatotropic viruses. At only 3,200 bp, HBV has one of the smallest genomes of all known pathogenic viruses. The partially double-stranded DNA (dsDNA) circular genome consists of four genes, X, Polymerase (P), Core (C), and Surface (S), and a high proportion of the genome is encoded on overlapping open reading frames (Figure 1). During transcription, the partially dsDNA genome is “completed” to form a fully dsDNA molecule, which is subsequently supercoiled to form covalently closed circular DNA (cccDNA). This cccDNA is reverse transcribed by HBV reverse transcriptase (RT), an enzyme lacking 3′–5′ exonuclease proof-reading capacity, and therefore introducing mutations into the HBV genome during each round of replication [in duck hepadnavirus, the mutation rate is estimated at between 0.8 × 10^−5^ and 4.5 × 10^−5^ substitutions per nucleotide per replication (2)]. The mutations generated result in a viral quasispecies, comprised of dominant genotype(s) surrounded by clouds of closely related HBV variants.

**Figure 2:**
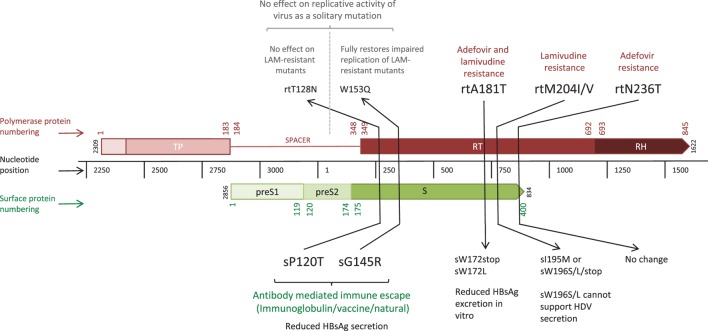
Linear depiction of overlapping reading frames of the Polymerase (P) and Surface (S) genes, highlighting five “mirror mutations” (4) where a nucleotide substitution influences amino acids in both S and P proteins. Gene lengths are given in nucleotides (black central bar); protein numbering based on the HBVdb X02763 amino acid sequence (7) is shown in color (Polymerase in red, Surface in green) for the whole protein. Mutations are given in amino acid positions in the affected gene segment, with the mutations in bold indicating the primary mutation with functional effect, and those in regular text indicating the secondary mirror mutation with any incidental functional effect.

The error prone RT, coupled with high rates of HBV replication [estimated at between 200 and 1,000 virions/hepatocyte/day at the peak of infection (3)] results in the production of a large number of virions harboring mutations. The vast majority of mutations are likely to be deleterious, some are neutral, and a minority provide the virus with a potential selective advantage, such as escape from CD8+ T cell-mediated responses. However, HBV polymorphisms are constrained by the overlapping reading frame structure of the genome, since the majority of mutations can simultaneously affect multiple genes [these have been described as “mirror” mutations (4), Figure 2]. Mutations that are neutral or beneficial for one protein might be detrimental for another. Accordingly, overlapping regions of the HBV genome generally have less diversity compared to non-overlapping regions (5) and the within-host rate of evolution at overlapping regions is about half that of non-overlapping regions (6).

Current vaccination and treatment approaches are hindered by poor diagnosis and access to treatment, drug and vaccine escape mutants, viral rebound on treatment cessation or immunosuppression, and lack of curative therapy (8). To make a significant impact on HBV prevalence, parallel improvements in diagnostics, treatment, and prevention are required; ultimately, new immunotherapeutic strategies may be key to the success of elimination. Developing a more robust picture of the extent, nature, and significance of the interplay between the virus and the host CD8+ T cell response is an important avenue of enquiry, enabling us to predict and tailor therapeutic interventions that may be beneficial in mediating control or clearance of chronic infection.

A robust body of data has been assimilated over the past few decades for HIV and HCV, informing significant understanding of the nature and impact of CD8+ T cell-mediated immune control and escape (Table 1). For HBV, there is a relative paucity of such evidence but the field could be advanced by similar approaches. We have therefore set out to assimilate the evidence for viral adaptation to the host CD8+ T cell response in HBV infection, and to consider the significance of this adaptation both to viral fitness and function, and to host outcomes. Finally, we highlight gaps in our current understanding and knowledge, in order to provide foundations for ongoing research efforts.

**Table 1:** Strands of evidence for the significance of the CD8+ T cell response in control/clearance of infection with blood-borne viruses.

Evidence for role of CD8+ T cell response in control of infection	HBV (details and citations^a^)	HCV (details and citations^a^)	HIV (details and citations^a^)
CD8+ T cell responses in acute infection associated with control and/or clearance	Functionally efficient, multi-specific antiviral CD8+ T cell responses (9)	CD8+ T cell responses are associated with clearance (10)	Inverse relationship between magnitude of acute CD8+ T cell response in acute infection and subsequent viral setpoint (11, 12)

*In vitro* killing assays demonstrate efficacy/potency of CD8+ T cell response	HBV-specific CD8+ T cells reduce viral loads in HBV-infected HepG2 (hNTCP) cells (13) Strong cytolytic response in woodchuck model (14)	*In vitro* system using replicons demonstrates HCV-specific CD8+ T cells strongly inhibit viral replication through cytolytic and non-cytolytic mechanisms in a dose-dependent manner (15) Rodent pegiviruses *in vitro* system shows promise (16)	CD8+ T cells targeting Gag show superior *in vitro* suppression of HIV replication (17)

Depletion of CD8+ T cell subsets impacts viremic control *in vitro and in vivo*	CD8^+^ depletion prolongs infection and delays viral clearance in chimpanzees (18)	Viral infection is prolonged in chimpanzees that previously cleared the infection after CD8+ T cells depletion (19)	Depletion of CD8+ T cell populations is associated with outgrowth of virus *in vitro* (20) and in the simian model *in vivo* (21)

Disease progression is associated with T cell exhaustion in chronic infection	Lack of protective T cell memory maturation and exhausted HBV-specific CD8+ T cell responses are seen in chronic infection (22–25) Blockade of inhibitory receptors PD-1 (26), CTLA-4 (27, 28), and Tim-3 (29) partly improve HBV-specific CD8+ T cell function *in vitro*	High PD-1 expression is associated with chronic infection (30)	PD-1 expression on HIV-specific T cells is associated with T cell exhaustion and disease progression (31)

CD8+ T cell responses may be required to maintain latency; viral mutations are associated with rebound or reactivation	Mutations identified in both neutralizing antibody targets and T cell epitopes are associated with reactivation (32–34)	Latency and reactivation are not typically associated with HCV infection	CD8+ T cell responses are associated with maintenance of latency, including during therapy, (35) and reviewed in Ref. (12) Persistence of escape mutations in viral reservoir can lead to reactivation (36)

Modulation of cell surface HLA expression as a mechanism of immune evasion	Variable surface expression of HLA is seen in human (37–39) and woodchuck hepatitis virus model (40)	Variable surface expression of class I and class II (41, 42)	HIV Nef is associated with downregulation of cell surface HLA expression (43, 44)

Evidence of efficacy of T cell vaccines	Heterologous prime-boost vaccines are promising (45, 46) Vaccine strategies assessed in woodchuck model (47)	Evidence from animal models (48) and human vaccine constructs are in development (49)	Vaccines expand the cellular immune response in rhesus monkeys (50) Reviewed in Ref. (51, 52)

HLA class I “footprints”—specific HLA alleles associated with viral polymorphisms identified, CD8+ T cell epitope escape and reversion occurs	Footprints identified in all four HBV genes (53–58) Further data are required to develop appropriate animal models (59)	Viral footprints in HLA epitopes display escape and reversion mutants identified (60–62)	CD8+ T cell escape mutations are associated with loss of viremic control; reversion to wild type is observed on transmission to an HLA-mismatched recipient [literature reviewed in Ref. (63)] SIV escape mutations selected in macaque model can influence disease outcome (64, 65)

Particular HLA class I alleles associated with disease control	HLA-A genotype is associated with HBeAg status (66) A database of HBV epitopes, “hepitopes,” highlights instances in which outcome is associated with specific T cell responses (53)	HLA-A*03 and HLA-B*27 alleles are protective in HCV infection (61, 67)	Strong association with HLA-B genotype (68); e.g., control epitomized by CD8+ responses restricted by HLA-B*57 and HLA-B*27 (69–71)

GWAS highlighting importance of HLA class I genes in control of chronic infection	Associations identified but mechanisms lacking (72–74). HLA-A*0301 is associated with clearance (75)	HLA-A*03, HLA-B*27 is associated with control (61, 76, 77)	Disease control is associated with SNPs in MHC region of human chromosome (78, 79), including specific association with HLA-B*57 expression

*^a^The citations within this table aim to provide a robust overview of the evidence, using a combination of strong examples from the primary literature together with selected review articles that summarize specific aspects of this topic*.

*HBV, hepatitis B virus; HBeAg, hepatitis B e antigen; HLA, human leukocyte antigen; GWAS, Genome wide association studies; SNPs, single-nucleotide polymorphisms; MHC, major histocompatibility complex; HCV, Hepatitis C virus; HIV, Human immunodeficiency virus*.

## The Immunological Basis for Escape

Acute and chronic HBV infections are associated with functionally different CD8+ T cell responses (Table 1). Acute, self-resolving infections are characterized by functionally efficient, multi-specific antiviral CD8+ T cell responses which are sustained after viral clearance (9). Both non-cytolytic and cytolytic mechanisms have been implicated (22). In contrast, chronic infection is typically characterized by a lack of protective T cell memory maturation and exhausted HBV-specific CD8+ T cell responses (22–24).

Th1-polarized CD4+ T cells regulate and maintain CD8+ T cell responses and contribute to HBV clearance (80). Genome wide association studies (GWAS) have linked a range of human leukocyte antigen (HLA) class II alleles with disease outcomes. CD4+ responses are associated with vaccine responses (81) and clearance of acute infection (82, 83). Host HLA class II genotype has also been linked to treatment response (84) and to risk of developing hepatocellular carcinoma (HCC) (85). CD4+ CD25+ regulatory T cells suppress the activation, proliferation, and interferon-γ production of both CD4+ and CD8+ T cells in chronic HBV infection (86, 87).

**Figure 3:**
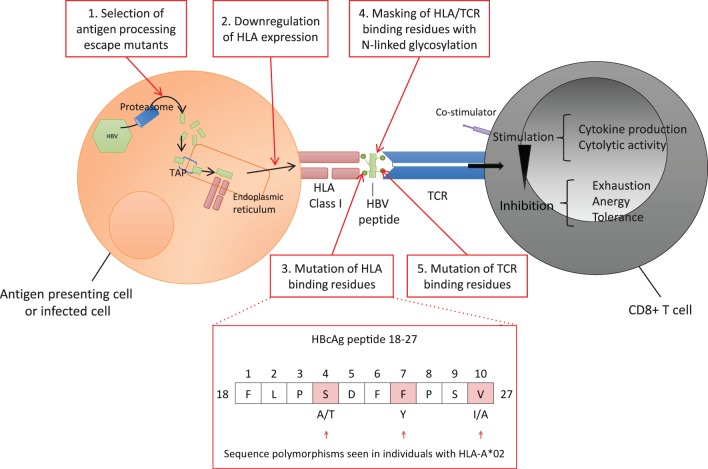
Hepatitis B virus (HBV) evasion of the host CD8+ T cell-mediated immune response. Viral peptides are processed into 7–12 mers by proteasomal degradation and are transported *via* the Transporter associated with Antigen Processing (TAP) into the endoplasmic reticulum. Peptides containing an appropriate motif are bound by human leukocyte antigen (HLA) class I molecules and transported to the cell surface for expression (92). Each T cell receptor (TCR) binds a range of specific HLA-peptide combinations. TCRs are concentrated on the cell surface over time at an “immunological synapse” triggering intracellular signaling (93). HBV can potentially escape the host CD8+ response at a number of points. 1. Evading antigen processing, 2. Downregulating presentation (37–40), 3. Altering HLA binding residues (54–56, 94), 4. Masking HLA epitope with N-linked glycosylation (NLG) sites (32, 95), 5. Altering TCR binding residues of the epitope (10, 96–99). Examples of polymorphic sites in HBV core antigen HLA-A*02 restricted FV10 epitope (residues 18–27) are highlighted (54).

The highly polymorphic HLA class I genes are thought to be an important host factor for viral control, contributing to differences in HBV outcome observed globally. Host HLA polymorphisms and different HBV genotypes have been demonstrated to influence the rate of disease progression and the long-term outcome of HBV infection (66, 88, 89). However, HBV can subvert various multiple steps of the CD8+ T cell antigen processing and presentation pathway to evade detection by the host (Figure 3, boxes 1–5). Thus, while all individuals with chronic HBV infection are at risk of increased progression to cirrhosis and HCC, individual outcomes depend on the interplay between host, viral, and environmental factors. In addition to HLA genes, other factors are implicated in disease outcome including age and duration of infection, other host genetic factors (90), and exposure to hepatotoxins (91).

## Mechanisms of HBV Escape from CD8+ T Cell Responses

### Antigen-Processing Escape Mutants

The amino acids flanking viral epitopes are important for effective antigen processing; mutations in these regions may impair proteasomal processing of the epitopes and are recognized in both HCV and HIV as a mechanism of CD8+ T cell escape (100, 101). Likewise, mutations altering the processing of HBV epitopes could be relevant for HBV escape from the CD8+ T cell-mediated immune response (Figure 3, box 1), however, none have been identified at present, potentially due to the focus on mutations lying within HLA-restricted epitopes rather than in the flanking regions.

### Virus Peptides Regulate Surface HLA Expression

Virus-induced changes in HLA class I surface expression play an important role in viral pathogenesis and persistence (Figure 3, box 2). CD8+ T cells recognize HBV-infected hepatocytes through presentation of HLA class I HBV epitopes on the cell surface (13), however, this expression can be upregulated or downregulated. Decreased presentation of class I MHC molecules on hepatocytes and lymphoid cells is described in the woodchuck hepatitis virus model (40). Changes in surface HLA have also been described in human HBV infection (37), for example, lower HLA class I has been associated with hepatitis B e antigen (HBeAg)-positive vs HBeAg-negative status (38, 39). Interestingly, these studies are three decades old and have not been replicated in the more recent literature. Downregulation of HLA class II molecules by pre-core mutants has also been described during chronic HBV infection (102). Mutations altering the processing or presentation of HBV HLA class I epitopes, although not conclusively demonstrated, could hypothetically be relevant for escape from the CD8+ T cell-mediated immune response in human infection (Figure 3, box 2).

### Selective Mutation of HLA-Binding Residues

Immune escape by selective mutation of HLA-binding residues within HBV CD8+ epitopes is one of the most commonly identified mechanisms for viral CD8+ immune escape (Table 1; Figure 3, box 3). Evidence of this escape mechanism in HBV has emerged through the identification of HLA class I “footprints” (94), mutations that are significantly enriched in patients with certain HLA class I alleles. Older literature was conflicting regarding the frequency and significance of such footprints in HBV (96, 103), but HLA footprints have subsequently been identified in all four HBV genes and mapped to known or predicted HLA epitopes [Table 1 (53)]. In some cases, these mutations result in altered peptide-HLA binding scores, providing a plausible mechanism for HBV immune escape, but have so far only been identified using cross-sectional data (54, 55, 96). However, the pattern of escape is consistent across populations with divergent HLA haplotypes and different HBV genotypes, for example, genotypes B and C in a cohort of Chinese-origin patients (56), New Zealand-resident Tongans with chronic HBV genotype C3 infections (57) and Iranian patients with genotype D infection (58). Importantly, core mutants in patients with chronic genotype A or D infection have been confirmed to impair CD8+ T cell IFN-γ secretion *in vitro* (54), indicating that these mutants could play an *in vivo* role in immune escape. These studies are limited by the sequencing methods (in which a pre-defined number of variants is typically selected for cloning and sequencing) reducing sensitivity for detection of low-abundance variant detection compared to newer ultra-deep sequencing methods.

Further studies have focused on identifying regions of the HBV genome with high within-host nucleotide diversity, and high rates of nonsynonymous substitutions, to determine which regions may be under strong HLA-mediated selection pressure. In a longitudinal study of eight HBeAg-negative asymptomatic HBV carriers, followed over 25 years, the ratio of synonymous to nonsynonymous mutations (dS/dN) in the core gene was low, suggesting high rates of positive selection, although no specific HLA-restricted epitopes were identified (104). Another study demonstrated low dS/dN ratios in patients with sustained vs unsustained viral control following treatment, with specific surface antigen polymorphisms lying within HLA class I epitopes identified [sV14G, sF20S, sT45I, sI213L (105)], although this was not confirmed either with HLA genotyping or through demonstration of a functional impact on T cell recognition.

### Epitope Masking With N-Linked Glycosylation (NLG)

N-linked glycosylation is a post-translational modification that plays an established role in the antigenicity and infectivity of viruses (106, 107). NLG can mask immunogenic epitopes, interfering with antibody recognition of hepatitis B surface antigen (HBsAg), leading to immune and diagnostic escape (32, 95). It can also impact on HBV virion secretion, likely by altering the ability of envelope proteins to interact with the capsid surface (108–110). The number of NLG sites correlates with disease state, with increased NLG reported in patients with reactivated HBV vs chronic infection, and in those with sustained vs unsustained response off treatment (32, 111). Although the role of NLG in HBV evasion of CD8+-mediated T cell immunity is yet to be determined, it could potentially provide another strategy for immune escape by interfering with the binding of an HBV epitope to an HLA molecule, or the binding of an HLA-antigen complex to a cognate T cell receptor (TCR) (Figure 3, box 4).

### Alteration of TCR Recognition

Engagement of the TCR with HLA class I/peptide complexes on antigen-presenting cells is key to activating CD8+ T cells; therefore, mutations in the TCR contact residues of an epitope can lead to immune escape. Immunodominance of viral epitopes is not simply determined by the amino acid sequence of the peptide and its binding affinity, but also depends on the peptide concentration and T cell clone, with the same HBV peptide able to induce different signaling cascades in different CD8+ T cell clones (97). Antagonist functions may provide HBV with a means of immune escape (Figure 3, box 5). Specifically, certain CD8+ T cell epitopes in hepatitis B core antigen (HBcAg) (97) and HBsAg (98) act as TCR antagonists, binding the TCR and inhibiting the CD8+ T cell response. HBeAg may promote HBV chronicity by inducing CD8+ T cell tolerance. However, the underlying mechanisms driving this immune state in humans remain to be elucidated. Indeed, the mechanism may not involve presentation of an HLA class I-restricted epitope, as currently no epitopes have been identified that are unique to the pre-core sequence of HBeAg (a ~29 amino acid stretch not shared with HBcAg) (112).

Chronic HBV infection is characterized by an exhausted CD8+ T cell phenotype associated with reduced cytotoxic activity and enhanced expression of inhibitory markers. TCR binding in the presence of high HBsAg levels induces T cell exhaustion, characterized by poor effector cytotoxic activity, impaired cytokine production and sustained expression of multiple inhibitory receptors. A hierarchy of co-inhibitory receptors, dominated by PD-1, act synergistically to promote CD8+ tolerance. The degree of T cell impairment also depends on suppressive cytokines, interaction with other T cell subsets, and stage of T cell differentiation (113–117). T cell exhaustion is (at least partly) reversible; blockade of inhibitory receptors including PD-1 (26, 117), CTLA-4 (27), and Tim-3 (29) partly improve HBV-specific CD8+ T cell function *in vitro*. In addition, therapy with nucleot(s)ide analogs may lead to a modest reconstitution of HBV-specific T cell function (118). Although this restoration is transient (119), these CD8+ T cells can be associated with viral control upon therapy cessation (120).

## Escape Over Space and Time

### Kinetics of Escape

Evidence of immune-mediated selection has been found in HBV infection (53–56), although the kinetics of immune escape are yet to be robustly delineated. Longitudinal samples from the same individuals form the ideal dataset to address questions about the changes in viral sequence and diversity over time, but this has rarely been undertaken for HBV infection. One longitudinal study of HBV evolution following acquisition from a single source demonstrated an expansion and contraction of HBV diversity, with maximum diversity coinciding with peak viremia, and a predominance of nonsynonymous mutations with greatest diversity in the core gene (3). Further longitudinal data are required to unpick the timing and kinetics of viral evolution.

An area of HBV kinetics that has received some attention is the scenario of HBeAg loss. It is hypothesized that the change from HBeAg-positive to HBeAg-negative occurs by one of two mechanisms:

(i) Antibody-mediated control (121, 122) usually associated with low HBV DNA levels. This situation is most likely to be characterized by low viral sequence diversity, although the low viral loads make this difficult to study given the limits of sensitivity of next generation sequencing approaches.
(ii) Selection of pre-core and promotor mutations (123), reducing or eliminating HBeAg production. In this case, HBeAg-negative status is associated with an increase in evolutionary rate and therefore with increased sequence diversity (6, 54, 56, 57, 104, 111, 124–126). The cause/effect relationship between the increased evolutionary rate and the shift in immune activity is unclear. The higher viral mutation rate could lead to the occurrence of stochastic mutations, generating new T cell epitopes that disrupt immune tolerance, or could be the consequence of increased immune reactivity driving escape mutants.

### Compartment-Specific Evolution

Compartment-specific evolution has been described for chronic viruses, including HIV (127, 128) and HCV (129, 130), although the evidence for HBV is very limited to date. The practical barriers to sampling tissue compartments longitudinally from the same patient make it difficult to assess the co-evolution of genetically distinct subpopulations over time [as is likely the case for HIV in the genital tract (131)].

Although *hepadnaviruses* are characteristically hepatotropic, HBV DNA is also found in a range of other tissues, including lymphatic cells. In the woodchuck model, life-long replication- and transmission-competent viruses persist in lymphocytes (132). However, it is difficult to demonstrate that hepatitis B virions isolated from different compartments in humans are replication and transmission competent, without a viable method of culturing autologous virus. There are some data to suggest that peripheral blood lymphocytes (PBLs) can support viral replication (133), but secretion of HBeAg and HBsAg from liver macrophages has not been detected [Lucifora, unpublished data, referred to in (134)].

HBV may undergo independent evolution in different tissue compartments, leading to compartmentalization of viral subpopulations (135–137); for example, HBV variants isolated from PBLs may be specifically adapted to this environment (137), potentially harboring relevant immune-escape mutants (135, 137). It has been hypothesized that compartment-specific mutants may serve as a source of reactivation or transmission and have been implicated in reinfection post liver transplant (138), mother to child transmission (137, 139), fulminant hepatic failure in the context of HIV co-infection (140), and antiviral escape (141).

Further work is required to confirm whether HBV does harbor replication and transmission competent viruses in cells other than hepatocytes. If this is confirmed, understanding host-virus dynamics at the compartmental level, studying the emergence of immune and antiviral escape mutants and the factors contributing to persistence and transmission will be crucial for developing improved therapeutics for HBV control.

## Functional Impact of Escape Mutations on HBV

The primary functional impact of mutations within HLA class I-restricted T cell epitopes is to alter the frequency and/or functionality of the CD8+ T cell immune response. These mutations may have additional impact on the viral replication cycle and treatment response in the following ways:

(i) Altered structure/function of the viral protein containing the mutated immune epitope (Figure 2). This is seen in HBV escape from B cell immunity in which mutations within the S gene are associated with diagnostic failure (HBsAg mutants are not detected by the immunological assay) and treatment failure due to changes in assembly and secretion, virion formation and HBV infectivity (109, 142, 143).
(ii) Effects of a “mirror” escape mutation on the overlapping gene (Figure 2). A mutation leading to amino acid substitutions in both the P and S genes, can simultaneously affect replicative capacity, drug resistance, and immunogenicity (142–145). Furthermore, deletions in regions such as the spacer region of Pol, neutral to Pol function, may lead to loss of immune epitopes in the overlapping preS1-preS2 region.
(iii) Impact of compensatory mutations mitigating for (i) and (ii). This is seen in replication deficient CD8+ immune escape mutants in HCV (146) and HIV (147, 148) but has not yet been identified in HBV.

The full range of functional impacts of HBV CD8+ immune escape mutants has not been comprehensively explored. Understanding the functional impact of mirror and compensatory mutations that are associated with CD8+ T cell-mediated selection may lead to further insights into the host–virus interaction.

## Clinical Impact of Virus and Host Polymorphisms on Host Outcome

### Impact of HBV Mutations on Reactivation

Hepatitis B virus reactivation as a consequence of immunosuppression has emerged as an important issue across a wide range of clinical settings [as previously reviewed (149, 150)]. Reactivation is seen secondary to immunosuppressive therapy for cancer, in particular in the context of therapy with rituximab and fludarabine (33, 151), solid organ transplantation (150), bone marrow transplantation (152), and autoimmune disease [especially with infliximab treatment (153–155)], highlighting that HBV reactivation is associated with a general defect of HBV-specific T cell control. Reactivation has also been documented in immunocompetent patients despite the presence of neutralizing antibodies (156).

Specific mutations associated with HBV reactivation have been identified in both neutralizing antibody targets and T cell epitopes (32–34). In a study of 29 patients with HBV reactivation, 75% of HBV-reactivated patients (vs 3% of chronic HBV controls) carried HBsAg mutations localized in immune-active HBsAg regions, and 5 of 13 identified HBsAg mutations were localized in HLA-restricted T cell epitopes [either class I (sC48G, sV96A, sL175S, and sG185E) or class II (sS171F)] (32). This suggests that in addition to an iatrogenic trigger for reactivation during immunosuppressive therapy, viral sequence can be a contributory factor as a result of CD8+ immune escape mutants.

### Impact of Host HLA Class I Haplotype on HBV Infection Outcome

GWAS approaches have linked various single-nucleotide polymorphisms in the HLA class II region with a range of infection outcomes, but there is a lack of such robust evidence for the involvement of HLA class I genes (72, 73, 75). One study identified a relationship between class I HLA-A genotype and HBeAg status (66), suggesting a role for genes at this locus in control of infection. However, confirmation of HLA associations can be difficult due to the variability in study design and methodologies and the small, heterogeneous populations sampled. Furthermore, the mechanisms for these HLA class I associations with disease outcome are poorly understood. Differences in antigen presentation, TCR binding leading to changes in T cell activation, and altered cytokine production may be responsible, either individually or in combination. Effects of linkage disequilibrium with other important neighboring loci, such as HLA class II or killer-cell immunoglobulin-like receptors genes, cannot be excluded. Functional studies are required to determine the basis for these associations.

### Impact of HBV Adaptation on Control Strategies

It is likely that to achieve elimination in line with global public health goals (157), new therapies targeting either the host immune system or the HBV replication cycle will be needed. Specific immunotherapies are under development, targeting both the innate and adaptive immune system, which aim to eliminate (or stably suppress) HBV replication (158).

T cell-based immune therapies are attractive options for HBV control (159). Strategies broadly take two approaches, either aiming to restore functionality and increase the quantity of existing defective host T cells with vaccines and checkpoint inhibitors, or to mimic the T cell response mounted during naturally resolving acute HBV infection by the adoptive transfer of HBV-specific T cells. Adoptive T cell therapy renders T cells HBV-specific by expression of natural HLA-restricted TCRs or HLA-independent chimeric antigen receptors on the T cell surface. Although natural TCRs have the advantage of activating the T cell response in a physiological way, therapy is potentially complicated by the need to match TCR to host HLA alleles, although some cross-reactivity may occur (160).

Given that HBV is able to evade natural immunity (32, 149, 156, 161, 162), vaccine-induced immunity (163, 164), and antiviral therapy (145, 165–169), it should be anticipated that HBV has the potential to mutate and escape from immunotherapeutic control. This is a vital consideration in the development of new HBV control strategies, and good knowledge of the full range of escape strategies should allow us to predict and potentially mitigate this. Care must be taken when developing T cell immunotherapies and polyepitope vaccines as immunodominance is a complex function of the nature and context of the epitope within the peptide, the TCR, the T cell clone, and the environment (98). The HBV literature is skewed toward the investigation of certain populations with specific HBV genotypes and HLA haplotypes, as highlighted in the “Hepitopes” database, a catalog of HLA class I epitopes in HBV, in which a disproportionate 44% of reported CD8+ T cell epitopes are HLA-A*02 restricted (53). The effect of using a polyepitope vaccine or re-directing T cells against peptides presented by discordant HLA alleles needs to be considered. This might inadvertently occur by using a vaccine or T cell-based immunotherapy based on key epitopes from a different genotype to that prevalent in the population to which it is delivered, and may produce functionally incompetent T cells, unable to recognize the infectious virus strain when used, or potentially lead to immunopathology. Since knowledge about certain host/virus interactions is under-represented, further studies will be required to define the full range of CD8+ T cell epitopes presented by HLA alleles, the antiviral functions of the corresponding CD8+ T cells in each compartment, the potential for generation of immune escape mutants and the impact these have on the immune response.

## Challenges

The understanding of HBV escape from the CD8+ immune response is lagging behind that of HIV and HCV. The field struggles with a lack of comprehensive literature, small datasets that can lead to conflicting results, differences in approaches to classifying patient groups into poor/outdated descriptions of “phases” of infection, over-reliance on serostatus, and lack of longitudinal follow-up and deep sequence data. Establishing the role of compartmentalization in infection is complex, with clinical samples scarce due to the risk associated with liver biopsy. These challenges are exacerbated by an under-resourcing of clinical and research approaches in many of the settings where HBV is endemic (8).

## Future Focus

There are many unanswered questions in the field of HBV and CD8+ immunity. In Table 2, we highlight gaps in our current understanding and knowledge, suggest desirable methods to develop, datasets to collate, and questions to be answered in order to provide foundations for ongoing research efforts.

**Table 2:** Areas for future focus in determining the nature and characteristics of the CD8+ T cell response to hepatitis B virus (HBV).

Approach	Rationale
Comprehensive case-finding and diagnostic strategy	To build a more complete picture of global HBV prevalence and distribution

Matched host genetic, clinical outcome and viral sequencing data, from populations with varying human leukocyte antigen (HLA) alleles and different infecting HBV genotypes, supported by improved case-finding strategy	To study the differential impact of viral and host genetics on host outcome. An unbiased approach is required to determine HLA genes that may be associated with specific clinical outcomes To investigate mechanisms behind reactivation in the immunosuppressed

Next generation sequencing of full-length HBV genome, including longitudinal deep sequencing data	To study the kinetics of viral transmission, evolution and escape, and the role played by viral quasispecies—higher sensitivity for low-abundance variant detection To identify novel mechanisms for CD8+ immune escape, e.g., antigen-processing escape mutants, regulation of HLA expression, N-linked glycosylation (Figure 3)

Culture systems for autologous HBV	To allow functional impact of patient-isolated HBV mutant strains to be studied, to determine fitness impact of the primary mutation, “mirror mutations” and compensatory mutations on replication, transmission, drug resistance immunogenicity, and clinical outcomes

Determination of 3D crystal structures for HBV proteins	To allow assessment of structural impact of viral polymorphisms, including consequences of immune and drug-mediated escape mutations

Comprehensive functional T cell studies	To understand how HLA class I escape mutants impact T cell function and the impact of viral and host genotype

Compartment-specific sampling to include liver and lymphoid tissue	To determine the presence of replication and transmission competent compartment-specific mutants and their dynamics including emergence of immune and antiviral escape mutants

## Summary

Hepatitis B virus is a complicated, unique virus, which has evolved together with *Homo sapiens* over millennia; it has evolved a range of mechanisms that favor transmission and persistence which include the capacity to evade the CD8+ T cell response. By focusing on understanding the evolutionary interplay between host and virus, we can develop better insights into areas where we can target viral “Achilles heels.” The need for novel anti-HBV strategies should drive a deeper exploration of this host–pathogen interaction. Future research will be strengthened by comprehensive cross-sectional and longitudinal studies on HLA-typed hosts with clinical details, across a range of host ethnicities and HBV genotypes, with high quality serological and whole genome HBV deep sequencing data. This will provide a more comprehensive understanding of the nature and mechanisms of HBV evolution and persistence, helping us to reach the goal of global HBV eradiation by guiding the design of new strategies, including vaccines and therapeutics.

## Author Contributions

SL undertook the primary literature review and drafted the manuscript; all authors had substantial input into revisions.

## Conflict of Interest Statement

The authors declare that the research was conducted in the absence of any commercial or financial relationships that could be construed as a potential conflict of interest. The handling Editor declared a past co-authorship with one of the authors PK.

## Abbreviations

C HBV, core gene; CARs, chimeric antigen receptors; cccDNA, covalently closed circular DNA; GWAS, genome wide association studies; HBV, hepatitis B virus; HBcAg, hepatitis B core antigen; HBeAg, hepatitis B e antigen; HBsAg, hepatitis B surface antigen; HCC, hepatocellular carcinoma; HLA, human leukocyte antigen; KIR, killer-cell immunoglobulin-like receptors; NLG, N-linked glycosylation; ORF, open reading frame; P HBV, polymerase gene; PBL, peripheral blood lymphocyte; RT, reverse transcriptase; S HBV, surface gene; SNP, single-nucleotide polymorphism; TCR, T cell receptor; MHC, major histocompatibility complex; HIV, Human immunodeficiency virus; HCV, Hepatitis C virus.
